# Comparative analyses of the prognosis, tumor immune microenvironment, and drug treatment response between left-sided and right-sided colon cancer by integrating scRNA-seq and bulk RNA-seq data

**DOI:** 10.18632/aging.204894

**Published:** 2023-07-24

**Authors:** Lichao Cao, Shenrui Zhang, Danni Yao, Ying Ba, Qi Weng, Jin Yang, Hezi Zhang, Yanan Ren

**Affiliations:** 1 Provincial Key Laboratory of Biotechnology of Shaanxi Province, Northwest University, Xi’an, China; 2 Key Laboratory of Resource Biology and Biotechnology in Western China, Ministry of Education, School of Life Sciences, Northwest University, Xi’an, China; 3 Shenzhen Nucleus Gene Technology Co., Ltd., Shenzhen, China

**Keywords:** colon cancer, scRNA-seq, prognosis, treatment, left-sided and right-sided

## Abstract

Background: In this study, we compared the prognosis, tumor immune microenvironment (TIM), and drug treatment response between left-sided (LCC) and right-sided (RCC) colon cancer to predict outcomes in patients with LCC and RCC.

Methods: Based on identified differentially expressed genes and using single-cell RNA sequencing data, we constructed and validated a prognostic model for LCC and RCC patients in the TCGA-COAD cohort and GSE103479 cohort. Moreover, we compared the differences of TIM characteristics and drug treatment response between LCC and RCC patients.

Results: We constructed and validated a five-gene prognostic model for LCC patients and a four-gene prognostic model for RCC patients, and both showed excellent performance. The RCC patients with higher risk scores were significantly associated with greater metastasis (*P* = 2.6×10^-5^), N stage (*P* = 0.012), advanced pathological stage (*P* = 1.4×10^-4^), and more stable microsatellite status (*P* = 0.007) but not T stage (*P* = 0.200). For LCC patients, the risk scores were not significantly associated with tumor stage and microsatellite status (*P* > 0.05). Additionally, immune infiltration by CD8 and regulatory T cells and M0, M1, and M2 macrophages differed significantly between LCC and RCC patients (*P* < 0.05). *APC* and *TP53* mutations were significantly more common in LCC patients (*P* < 0.05). In contrast, *KRAS*, *SYNE1*, and *MUC16* mutations were significantly more common in RCC patients (*P* < 0.05). In addition, tumor mutation burden values were significantly higher in RCC patients than in LCC patients (*P* = 5.9×10^-8^). Moreover, the expression of immune checkpoint targets was significantly higher in RCC patients than in LCC patients (*P* < 0.05), indicating that RCC patients maybe more sensitive to immunotherapy. However, LCC and RCC patients did not differ significantly in their sensitivity to eight selected chemicals or target drugs (*P* > 0.05). The average half-maximal inhibitory concentrations for camptothecin, teniposide, vinorelbine, and mitoxantrone were significantly lower in low-risk than in high-risk RCC patients (*P* < 0.05), indicating that the lower risk score of RCC patients, the more sensitive they were to these four drugs.

Conclusions: We investigated the differences in prognosis, TIM, and drug treatment response between LCC and RCC patients, which may contribute to accurate colon cancer prognosis and treatment of colon cancer.

## INTRODUCTION

Colon cancer (CC) is a common gastrointestinal malignant tumor with high mortality worldwide [1]. Numerous studies have identified various prognostic signatures for CC [2–4] to facilitate CC prognosis and treatment. However, the tumor heterogeneity greatly complicates the treatment and prognosis of CC patients. A landmark study has divided CC into four consensus molecular subtypes (CMS) using aggregated gene expression data and facilitate the translation of molecular subtypes into the clinic [5]. Otherwise, CC can be subdivided into left-sided (LCC) and right-sided (RCC) based on the colon’s anatomical structure [6]. However, the tumor microenvironment (TME), prognosis, and treatment of LCC and RCC patients are inconsistent [7–9]. Therefore, exploring the potential relationship between CMS subtypes and RCC-LCC subtypes and exploring the heterogeneity may be critical for improving precision treatment of CC.

Single-cell next-generation RNA sequencing (scRNA-seq) can be used to investigate gene expression profiles at the single-cell level, facilitating the dissection of previously hidden heterogeneity in cell populations [10]. Therefore, exploring crucial genes based on scRNA-seq data could provide more meaningful prognostic signatures and drug treatment for CC. A recent study identified ferroptosis-related subtypes, investigated LCC and RCC heterogeneity, and established a scoring model to quantify tumor immune microenvironment (TIM) characteristics [11]. However, few studies have explored TME, prognosis, and treatment differences between LCC and RCC patients using scRNA-seq data.

By combining scRNA-seq data and bulk RNA-seq data, our study established and verified the prognostic models for LCC and RCC patients, and compared their differences of prognosis, microenvironment and drug treatment response. Our findings may facilitate more accurate predictions of the prognosis and potential treatment benefits for CC patients.

## MATERIALS AND METHODS

### Data download

The scRNA-seq dataset GSE200997, comprising 7 normal and 15 tumor samples, was downloaded from the US National Center for Biotechnology Information’s Gene Expression Omnibus (GEO) database (https://www.ncbi.nlm.nih.gov/geo/). The original data included 23,828 genes and 49,859 cells, of which 18,273 cells were from normal samples, and 31,586 were from tumor samples. And the cells from tumor samples were used for further analysis, of which 16,448 cells were LCC cells and 15,138 were RCC cells.

And another scRNA-seq dataset, GSE144735, was also downloaded from GEO database for verification. The dataset included 27,414 cells from 6 patients in the core and border regions, as well as in matched normal mucosa. Based on a previously reported study [7], the cecum, ascending, and hepatic belong to the right side, and the splenic, descending, sigmoid, rectum, and junction belong to the left side. Finally, 5,780 LCC cells and 2,474 RCC cells from the core region of tumor samples were selected for subsequent validation analysis.

The Cancer Genome Atlas Colon Adenocarcinoma (TCGA-COAD) bulk RNA-seq data were downloaded from the University of California-Santa Cruz Xena platform (https://xenabrowser.net/datapages/). Only samples with survival and location information were included. Similarly, based on the published literature [7], we selected 394 samples, including 149 LCC samples, 224 RCC samples, and 21 transverse sectional samples. Their detailed information is provided in Table 1. Furthemore, we also downloaded their corresponding somatic mutation profiling data.

**Table 1:** Detailed information of TCGA-COAD cohort.

	**Left**	**Right**	**Transverse**	**Overall**
	**(N=149)**	**(N=224)**	**(N=21)**	**(N=394)**
**Gender**				
female	79 (53.0%)	102 (45.5%)	13 (61.9%)	194 (49.2%)
male	70 (47.0%)	122 (54.5%)	8 (38.1%)	200 (50.8%)
**Age**				
<=60	51 (34.2%)	55 (24.6%)	12 (57.1%)	118 (29.9%)
>60	98 (65.8%)	169 (75.4%)	9 (42.9%)	276 (70.1%)
**Microsatellite status**				
N/A	4 (2.7%)	7 (3.1%)	0 (0%)	11 (2.8%)
Indeterminate	3 (2.0%)	0 (0%)	0 (0%)	3 (0.8%)
MSI-H	7 (4.7%)	65 (29.0%)	4 (19.0%)	76 (19.3%)
MSI-L	26 (17.4%)	34 (15.2%)	4 (19.0%)	64 (16.2%)
MSS	109 (73.2%)	118 (52.7%)	13 (61.9%)	240 (60.9%)
**AJCC-Stage**				
i	23 (15.4%)	39 (17.4%)	1 (4.8%)	63 (16.0%)
ii	49 (32.9%)	92 (41.1%)	8 (38.1%)	149 (37.8%)
iii	49 (32.9%)	60 (26.8%)	9 (42.9%)	118 (29.9%)
iv	25 (16.8%)	27 (12.1%)	1 (4.8%)	53 (13.5%)
Missing	3 (2.0%)	6 (2.7%)	2 (9.5%)	11 (2.8%)
**AJCC-T**				
T1	5 (3.4%)	4 (1.8%)	1 (4.8%)	10 (2.5%)
T2	26 (17.4%)	38 (17.0%)	0 (0%)	64 (16.2%)
T3	108 (72.5%)	156 (69.6%)	15 (71.4%)	279 (70.8%)
T4	10 (6.7%)	25 (11.2%)	5 (23.8%)	40 (10.2%)
Missing	0 (0%)	1 (0.4%)	0 (0%)	1 (0.3%)
**AJCC-N**				
N0	76 (51.0%)	139 (62.1%)	11 (52.4%)	226 (57.4%)
N1	48 (32.2%)	44 (19.6%)	7 (33.3%)	99 (25.1%)
N2	25 (16.8%)	41 (18.3%)	3 (14.3%)	69 (17.5%)
**AJCC-M**				
M0	103 (69.1%)	163 (72.8%)	17 (81.0%)	283 (71.8%)
M1	25 (16.8%)	27 (12.1%)	1 (4.8%)	53 (13.5%)
MX	19 (12.8%)	28 (12.5%)	3 (14.3%)	50 (12.7%)
Missing	2 (1.3%)	6 (2.7%)	0 (0%)	8 (2.0%)

Abbreviation: AJCC, American Joint Committee on Cancer.

The independent dataset GSE103479 was downloaded from GEO database (https://www.ncbi.nlm.nih.gov/geo/), which included 77 LCC and 59 RCC samples, detailed information could be seen in Supplementary Table 1.

### Identification of the location-related differentially expressed genes (DEGs) based on the dataset GSE200997

The various functions in *Seurat* package (version=4.1.1) for the R statistical software (v.4.0.2; http://www.R-project.org) was used to analyze the scRNA-seq data [12–14]. Genes expressed in <3 cells and cells expressing <50 genes or >6,000 genes were excluded. In addition, the mitochondrial content was <20%.

The filtered data were normalized using the *NormalizeData* function, and the first 3000 highly variable genes were screened using the *FindVariableFeatures* function. Next, the features were scaled using the *ScaleData* function, and their dimensionality was reduced using the *RunPCA* function. Then, we set dim = 20 and clustered the cells using the *FindNeighbors* and *FindClusters* functions. Importantly, we conducted the t-distributed stochastic neighbor embedding and uniform manifold approximation and projection (UMAP) algorithms to reduce dimensionality further and visualize the cluster classification based on the selected top 20 principal components. In addition, the cell types of clusters were annotated using R’s *SingleR* package, with *HumanPrimaryCellAtlasData* as the reference [15]. Moreover, we identified DEGs between LCC and RCC cells in each cell type using the *FindMarkers* function with logfc.threshold = 0.585. Finally, we identified location-related DEGs for CC by merging the DEGs identified in all annotated cell types. Additionally, location-related DEGs were subjected to gene ontology (GO) functional and Kyoto Encyclopedia of Genes and Genomes (KEGG) enrichment analyses using R’s *clusterProfiler* package [16].

### Prognostic LCC and RCC signature identification and validation

The tumor samples containing expression data and survival information in the TCGA-COAD cohort were used to establish the prognostic model. Finally, 123 LCC samples and 185 RCC samples were included. Kaplan–Meier survival and univariate Cox regression analyses were performed on the location-related DEGs in the LCC and RCC expression profile data with a cutoff criterion of *P* < 0.05. The genes with maximum prognostic value were selected for least absolute shrinkage and selection operator (LASSO) regression analysis. Next, we established separate risk score models predicting LCC and RCC patient prognosis using a multivariate Cox regression analysis. Then, each patient’s risk score was calculated according to the following formula:


$$\begin{array}{l} risk\;score\\ =\sum_{i}^{n}coefficient\;of\;\chi i\\ \times \;scaled\;expression\;value\;of\;\chi i \end{array}$$

where “*χi*” represents the current signature, “i” represents the location of the current signature, and “n” represents the number of the whole signatures. Moreover, the prognostic signatures for LCC and RCC patients were validated using an independent cohort GSE103479.

Additionally, we investigated the expression of these prognostic genes in different LCC and RCC cell types of scRNA-seq datasets (GSE200997 and GSE14473), and compared the expression differences in LCC and RCC samples of bulk RNA-seq datasets (TCGA-COAD cohort and GSE103479).

### The CMS analysis

We predicted the CMS group of each tumor sample of TCGA-COAD cohort using R’s *CMScaller* package [17], the samples with an *FDR* > 0.05 were filtered out. Then, we calculated the proportion of LCC and RCC samples in each CMS group, and compared the relationship between each CMS group and OS. Moreover, the expression differences of the prognostic markers among CMS groups were compared using *Kruskal–Wallis* test.

### Statistical analysis

We compared the overall survival (OS) status between LCC and RCC patients in the TCGA-COAD cohort and GSE103479 cohort by using the Kruskal–Wallis test. Additionally, receiver operating characteristic (ROC) and Kaplan–Meier curves were plotted to assess the predictive capabilities of the established risk score prognostic models. The most significant difference between true and false positive points on the ROC curve was selected as the best critical value for grouping patients. Moreover, we evaluated the performance of prognostic models in predicting tumor staging and microsatellite status for LCC and RCC patients. All results with *P* < 0.05 were considered statistically significant.

### Exploring TIM characteristics in LCC and RCC patients

Consistent with the samples used in constructing LCC and RCC prognosis models, the CIBERSORT algorithm was used to estimate the proportion of 22 tumor infiltrating immune cell types in each sample based on expression profiling dataset [18]. Differences in the immune landscape between the LCC and RCC patients were assessed using an unpaired *t*-test. The *cor.test* function in R was used to assess the correlations between the estimated proportions of immune cell types and the risk score of each sample in LCC and RCC patients. Moreover, we compared the mRNA levels of immune checkpoint proteins and their ligands between LCC and RCC patients and high-risk and low-risk patients.

Additionally, tumor samples containing somatic mutation data and survival information in the TCGA-COAD cohort were used to explore the mutation characteristics of LCC and RCC samples. Finally, 102 LCC samples and 166 RCC samples were included. First, we analyzed and visualized LCC and RCC patients’ somatic mutation profiles using the *maftools* R package [19]. Genes with significant mutation differences between LCC and RCC patients were assessed for their association with OS. Moreover, the fraction of affected oncogenic pathways was predicted using the *OncogenicPathways* function in the *maftools* R package. Next, we calculated the tumor mutation burden (TMB) value and visualized the LCC and RCC patient mutation profiling dataset. Then, TMB values and mutation profiles were compared between LCC and RCC patients. The optimal cutoff value of TMB was determined by the *surv_cutpoint* algorithm of *survivial* R package and then all samples were categorized into TMB-high group and TMB-low group. Subsequently, Kaplan–Meier curves were plotted to visualize the association between TMB values and OS in LCC and RCC patients. Finally, we calculated the median value of the sample’s TMB, and the samples with TMB values higher than this median value were classified as TMB-high group, while the samples with TMB values lower than this median value were classified as TMB-low group. Subsequently, the relationship between TMB and OS were further analyzed. All results with *P* < 0.05 were considered statistically significant.

### Prediction and comparison of drug sensitivity in LCC and RCC patients

Consistent with the samples used in constructing LCC and RCC prognosis models, and based on the expression profile dataset of these samples, we predicted the half-maximal inhibitory concentration (IC_50_) of cancer drugs in LCC and RCC patients using the *oncoPredict* R package [20] and selected drugs with an average IC_50_ <5 and related them to CC treatment. Next, we compared responses to these drugs in LCC and RCC patients and high-risk and low-risk LCC and RCC patients.

### Data availability statement

The TCGA-COAD cohort was available in the UCSC Xena (https://xenabrowser.net/datapages/). The scRNA-seq datasets GSE200997, GSE14473 and bulk RNA-seq data GSE103479 were download from NCBI-GEO database (https://www.ncbi.nlm.nih.gov/geo/).

## RESULTS

### Location-related DEGs identification in different cell types

**Figure 1:**
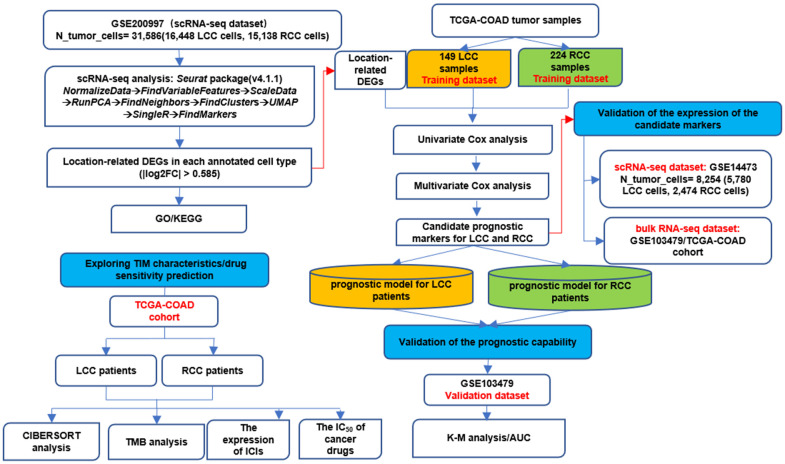
The whole analysis workflow.

**Figure 2:**
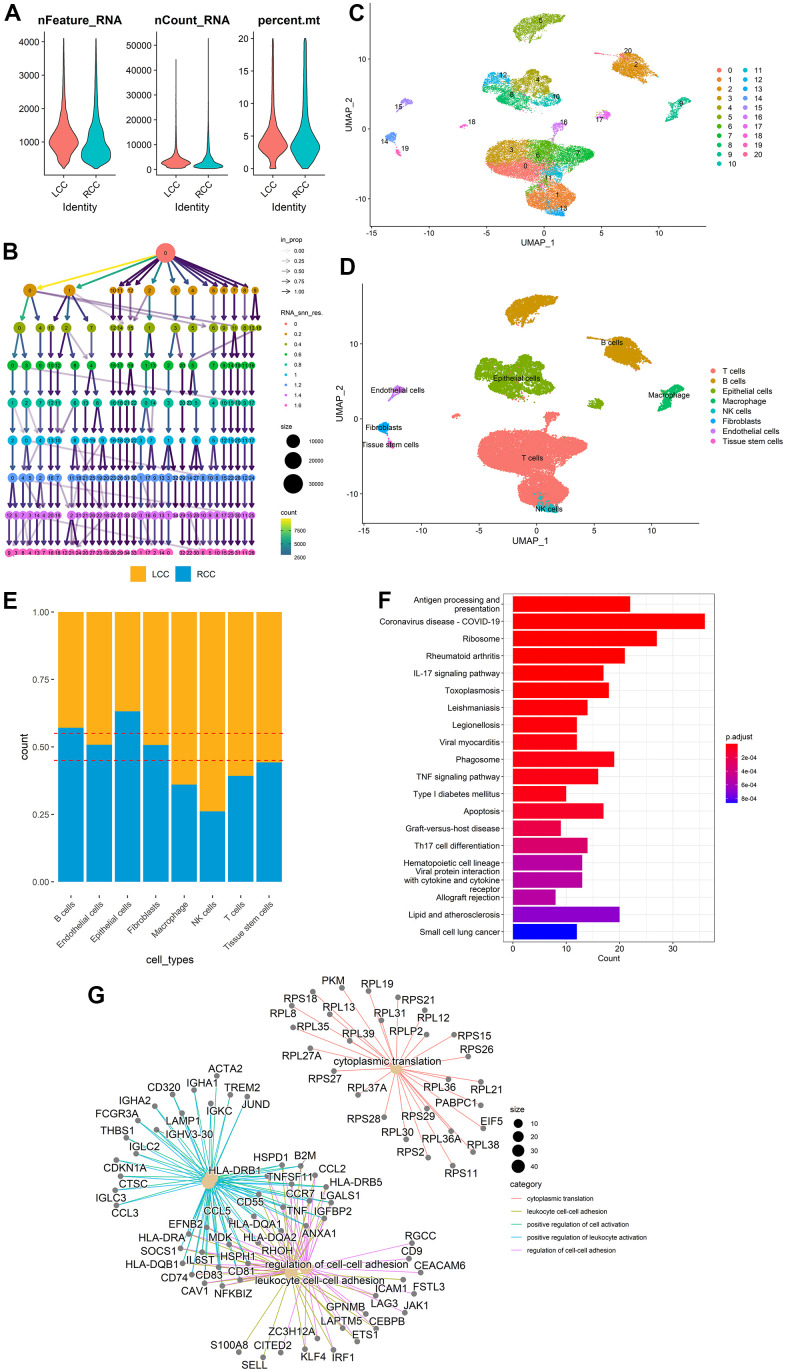
**DEG identification between LCC and RCC in different cell types from scRNA-seq data.** (**A**) scRNA-seq data quality control for LCC and RCC cells. (**B**) A cluster tree with a resolution range of 0 to 1.6. (**C**) All cells were classified into 21 clusters using the UMAP algorithm. (**D**) The clusters were annotated into eight main cell types using the *singleR* R package. (**E**) LCC and RCC distributions for each annotated cell type. (**F**) DEGs KEGG analysis results. (**G**) DEGs GO analysis results.

Figure 1 highlights the whole analysis workflow. After filtering according to the selection criteria, there were 31,586 tumor cells in the scRNA-seq dataset (GSE200997), of which 16,448 were from LCC patients and 15,138 were from RCC patients. The quality control data of scRNA-seq data, such as the range of RNA features, counts, and mitochondrial gene expression percentages for each cell, were shown in Figure 2A. The cluster tree with a resolution range of 0 to 1.6 showed that when RNA_ Snn_ was equal to 0.6, the number of branches was minimized, making it the optimal choice for dimensionality reduction (Figure 2B). Then, all cells were classified into 21 clusters using the UMAP algorithm and further automatically annotated into eight main cell types using the *singleR* R package (Figure 2C, 2D). LCC and RCC cell distributions in each annotated cell type were shown in Figure 2E. LCC and RCC DEGs statistics in each annotated cell type were provided in Table 2. More detailed DEGs information was provided in Supplementary Table 2. Finally, 690 location-related DEGs were obtained by merging the DEGs identified from each cell type. Functional and pathway enrichment analyses were performed on the location-related DEGs using the *clusterProfiler* R package. KEGG analysis indicated that the location-related DEGs were significantly enriched for terms associated with the interleukin-17 and tumor necrosis factor signaling pathways and apoptosis (Figure 2F). GO analysis indicated they had functions related to cytoplasmic translation, positive cell activation regulation, and cell-cell adhesion regulation (Figure 2G).

**Table 2:** The statistics of DEGs between LCC and RCC in each annotated cell type.

**Cell type**	**Cluster number**	**Up-regulated genes**	**Down-regulated genes**	**All genes**
T cells	0,1,3,5,6,7,11,16	62	14	76
B cells	2,5,20	122	27	149
Epithelial cells	4,8,10,12,17	81	60	141
NK cells	13	104	26	130
Macrophage	9	40	22	66
Fibroblasts	14	66	62	128
Endothelial cells	15	33	33	66
Tissue stem cells	19	139	108	247

### Construction and validation of prognostic models for LCC or RCC patients

Based on TCGA-COAD cohort, we observed that the survival time was significantly longer in LCC patients than in RCC patients (*P* = 0.038; Supplementary Figure 1A). However, survival time did not differ significantly between patients with CC in the transverse section and LCC (*P* = 0.830) or RCC (*P* = 0.530) patients. In the GSE103479 cohort, the prognosis of LCC patients was also significantly better than that of RCC patients (*P* =0.043, Supplementary Figure 1B). In TCGA-COAD cohort, the relationship between CMS and OS was not significant (*P* >0.05, Supplementary Figure 1C). In GSE103479 cohort, CMS3 had the best OS, while CMS1 had the worst OS (Supplementary Figure 1D).

The Kaplan–Meier survival analysis identified 17 genes significantly associated with LCC patient prognosis and 20 genes significantly associated with RCC patient prognosis (*P* < 0.05; Supplementary Table 3). Univariate Cox regression analysis indicated that five were independent indicators for LCC patients and eight for RCC patients (Table 3). After further shrinkage through LASSO regression, we selected five genes for LCC patients and four for RCC patients. The coefficient of each prognostic signature was shown in Supplementary Figure 1E, 1F. The Kaplan-Meier curves of those genes were shown in Supplementary Figure 2.

**Table 3:** The detailed information of the potential independent signatures for patients with LCC or RCC.

**Patients**	**Prognostic signatures**	**HR**	**HR.95L**	**HR.95H**	**p.value**
**RCC**	HSPA1A	1.41356217671282	1.16350097826825	1.71736686496571	0.00049297581800357
	FOS	0.81584830453234	0.673878317257311	0.987727960616577	0.0369283627502978
	CD69	0.78042371991399	0.641390931256157	0.949594315921415	0.0132626664542913
	RHOH	0.800002515242927	0.64806037197813	0.987568523039716	0.0378600748868144
	KLF4	0.775803574145191	0.607680844919644	0.99043962087704	0.0416450641807661
	TFF1	0.905647012239849	0.823929582435838	0.995469186036703	0.039967568997509
	GDF15	0.622015292962873	0.495507478853596	0.780821765949578	0.0000426660693411279
	LGALS2	0.833641956980532	0.705030165125973	0.985715146406761	0.0333158089764621
					
**LCC**	FOSB	1.38792045878328	1.12035352253476	1.71938871183352	0.00269970420423281
	RPL35	0.503130789685977	0.303313150461264	0.834584953356195	0.00780834395123433
	REG1A	0.88427652277397	0.798529419282176	0.979231259171564	0.0181153181968568
	TESC	0.774472331024749	0.604301611845149	0.992562951621926	0.0434941395308241
	C11orf96	1.51919402433543	1.05004983019869	2.1979437710492	0.0264780661405852

Abbreviation: HR, hazard ratio.

**Figure 3:**
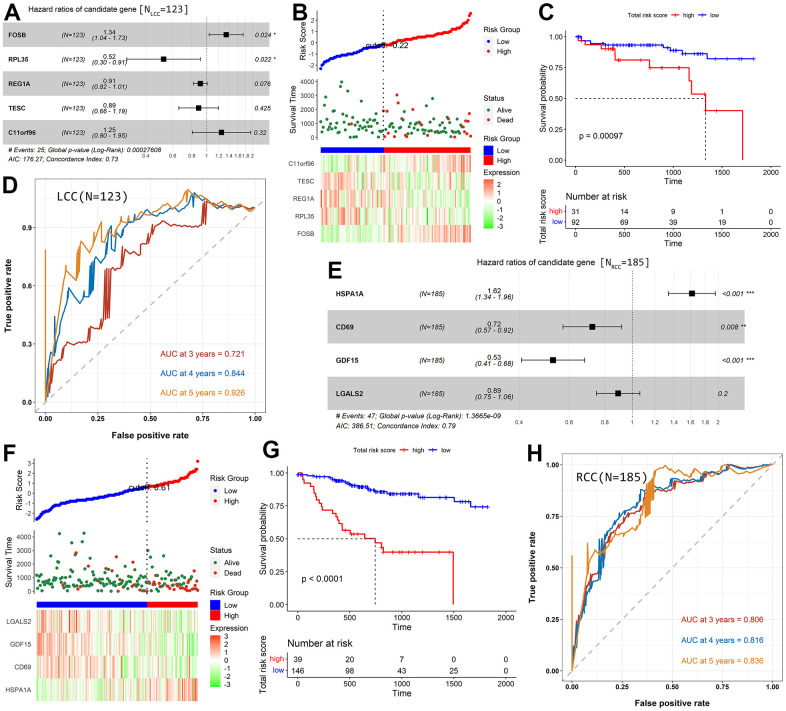
**Construction and evaluation of prognostic models for LCC and RCC patients based on TCGA-COAD cohort.** (**A**) A Forest plot shows the HR value of each candidate prognostic gene for LCC patients. (**B**) The distribution of LCC patients in the high- and low-risk score groups and their relationship with OS and the expression pattern of five prognostic genes. (**C**) A Kaplan–Meier curve shows that LCC patient OS was significantly higher in the low-risk score group than in the high-risk score group. (**D**) The AUCs of the prognostic model for LCC patients. (**E**) A Forest plot showing the HR value of each candidate prognostic gene in RCC patients. (**F**) The distribution of RCC patients in the high- and low-risk score groups and their relationship with OS and the expression pattern of four prognostic genes. (**G**) A Kaplan–Meier curve shows that RCC patient OS was significantly higher in the low-risk score group than in the high-risk score group. (**H**) The AUCs of the prognostic model for RCC patients.

In the prognostic model for LCC patients, two genes (FosB proto-oncogene AP-1 transcription factor subunit [*FOSB*] and chromosome 11 open reading frame 96 [C11orf96]) were risk factors (hazard ratio [HR] >1), while three (ribosomal protein L35 [*RPL35*], regenerating family member 1 alpha [*REG1A*], and tescalcin [*TESC*]) were protective factors (HR <1; Figure 3A). The heatmap showed that the risk factors were downregulated in the low-risk group and upregulated in the high-risk group (Figure 3B). In contrast, the protective factors showed the opposite pattern. The scatter diagram in Figure 3B indicated that the OS of patients was better in the low-risk group than in the high-risk group, consistent with the results in Figure 3C (*P* = 9.7×10^-4^). The areas under the ROC curve (AUC) for the prognostic model at 3-, 4-, and 5-year OS are 0.721, 0.844, and 0.926, respectively (Figure 3D).

There were four genes in the prognostic model for RCC patients. One (heat shock protein family A member 1A [*HSPA1A*]) was a risk factor (HR >1), and three (cluster of differentiation 69 [*CD69*], growth differentiation factor 15 [*GDF15*], and galectin 2 [*LGALS2*]) were protective factors (HR <1; Figure 3E). Consistent with the prognostic model for LCC patients, we found that the expression of risk factor was low in low-risk patients and high in high-risk patients. In contrast, the protective factors showed the opposite pattern (Figure 3F). In addition, patients in the low-risk group had better prognoses than those in the high-risk group (*P* < 1.0×10^-4^; Figure 3G). The model’s AUCs were 0.806 for 3-year OS, 0.816 for 4-year OS, and 0.836 for 5-year OS (Figure 3H).

We further validated the effectiveness of the identified prognostic markers based on GSE103479 cohort. Consistent with the results obtained in the prognosis model based on TCGA-COAD cohort, in the LCC prognosis model, the prognosis of patients with low-risk patients was significantly better than that of patients with low-risk score (*P* = 0.014, Supplementary Figure 3A), and the AUC for the prognostic model at 3-, 4-, and 5-year OS are 0.680, 0.688, and 0.615, respectively (Supplementary Figure 3B). In the RCC prognosis model, the low-risk patients’ OS was significantly longer than high-risk patients’ OS (*P* =0.012, Supplementary Figure 3C), and the model’s AUCs were 0.615 for 3-year OS, 0.710 for 4-year OS, and 0.639 for 5-year OS (Supplementary Figure 3D).

**Figure 4:**
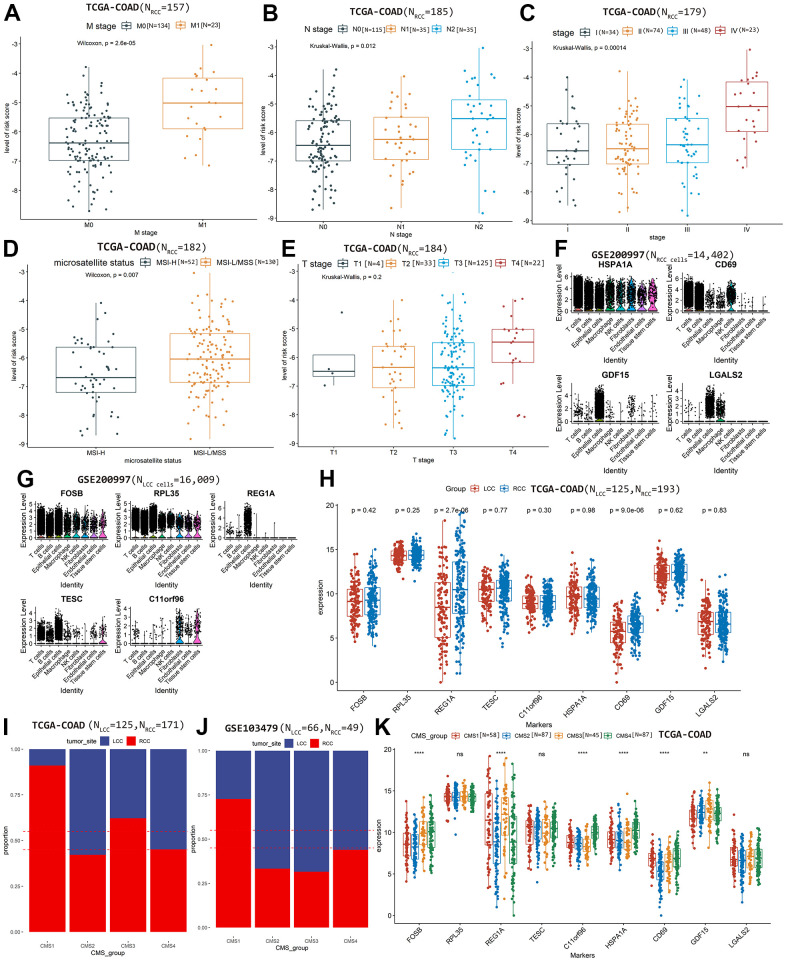
The relationship between the risk score of RCC prognostic model and clinical features, (**A**) M stage, (**B**) N stage, (**C**) Advanced pathological stages, (**D**) Microsatellite status, (**E**) T stage. The prognostic markers’ expression level in each cell types based on scRNA-seq dataset GSE200997, (**F**) RCC cells, (**G**) LCC cells. (**H**) Comparing the differences of markers’ expression between LCC and RCC samples based on TCGA-COAD cohort. Comparing the proportions of LCC and RCC patients in each CMS group on TCGA-COAD cohort (**I**) and GSE103479 (**J**). (**K**) Comparing the expression differences of markers among CMS groups based on TCGA-COAD cohort.

We also evaluated the performance of prognostic model in predicting tumor staging and microsatellite status based on TCGA-COAD cohort. In the RCC prognostic model, higher risk scores were significantly associated with greater metastasis (*P* = 2.6×10^-5^; Figure 4A), N stage (*P* = 0.012; Figure 4B), advanced pathological stage (*P* = 1.4×10^-4^; Figure 4C), and more stable microsatellite status (*P* = 0.007; Figure 4D) but not T stage (*P* = 0.200; Figure 4E). However, in the LCC prognostic model, risk scores were not significantly associated with tumor stage and microsatellite status (*P* >0.05; Supplementary Figure 3E–3I), potentially reflecting the small sample size at a certain level (<30; Table 1), leading to no statistical significance.

### The expression of the candidate prognostic genes in scRNA-seq and bulk RNAseq datasets

Based on scRNA-seq dataset GSE200997, the differential expression of the marker genes between LCC cells and RCC cells in each annotated cell type was shown in Table 4. We observed that *HSPA1A* was expressed in all RCC cell types, *CD69* was mainly expressed in immune-related cell types, including T, B, and natural killer cells; while *GDF15* was mainly expressed in epithelial cells, and *LGALS2* was expressed in epithelial cells and macrophages (Figure 4F). *FOSB* and *RPL35* were expressed in all LCC cell types; while *REG1A* was expressed in epithelial cells; *TESC* was mainly expressed in T, B, and epithelial cells; and *C11orf96* was mainly expressed in fibroblasts and tissue stem cells (Figure 4G).

**Table 4:** The differential expression of the marker genes between LCC cells and RCC cells in each cell type based on GSE200997.

**Markers**	**myAUC**	**avg_diff**	**Power**	**avg_log2FC**	**pct.1**	**pct.2**	**Celltypes**
*FOSB*	0.599	0.4618231	0.198	0.666269896	0.562	0.373	T Cells
*HSPA1A*	0.824	2.278156184	0.648	3.286684629	0.752	0.154	T Cells
*FOSB*	0.637	0.662858049	0.274	0.95630202	0.579	0.361	B Cells
*HSPA1A*	0.776	2.163085844	0.552	3.120673221	0.635	0.118	B Cells
*CD69*	0.682	1.277230084	0.364	1.842653508	0.642	0.374	B Cells
*FOSB*	0.58	0.423361877	0.16	0.61078208	0.593	0.481	NK cells
*HSPA1A*	0.818	2.235335966	0.636	3.224908112	0.704	0.084	NK cells
*HSPA1A*	0.565	0.447071753	0.13	0.644988201	0.664	0.601	macrophage
*CD69*	0.564	0.622492906	0.128	0.898067428	0.216	0.092	macrophage
*RPL35*	0.645	0.579500519	0.29	0.836042525	0.854	0.789	epithelial
*REG1A*	0.431	-1.121210588	0.138	-1.617564954	0.03	0.168	epithelial
*TESC*	0.436	-0.676260355	0.128	-0.97563746	0.15	0.264	epithelial
*GDF15*	0.565	0.652976527	0.13	0.942045997	0.521	0.455	epithelial
*LGALS2*	0.442	-0.901389298	0.116	-1.30042987	0.05	0.162	epithelial
*C11orf96*	0.571	0.454403273	0.142	0.655565348	0.46	0.362	fibroblasts
*HSPA1A*	0.388	-0.843405708	0.224	-1.216777233	0.684	0.751	fibroblasts
*GDF15*	0.424	-0.562241804	0.152	-0.811143462	0.107	0.258	fibroblasts
*FOSB*	0.403	-0.434459647	0.194	-0.626792779	0.422	0.581	endothelial
*C11orf96*	0.566	0.503324559	0.132	0.726143845	0.286	0.167	endothelial
*HSPA1A*	0.373	-0.646403691	0.254	-0.932563399	0.656	0.747	endothelial
*FOSB*	0.388	-0.481429936	0.224	-0.694556582	0.382	0.617	stem cells
*C11orf96*	0.561	0.527176636	0.122	0.760555119	0.52	0.469	stem cells
*HSPA1A*	0.31	-1.093683253	0.38	-1.577851406	0.598	0.815	stem cells

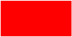
 The red color means markers for LCC.

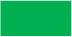
 The green color means markers for RCC.

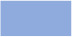
 The cyan color means type1 cells.

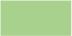
 The lawngreen color means type2 cells.

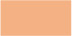
 The brown color means type3 cells.

Based on another scRNA-seq dataset GSE14473, we validated the expression differences of the candidate markers between LCC and RCC cells, and we found that all candidate markers were significantly differentially expressed except for *C11orf96* (Table 5). Moreover, we also observed the expression patterns of the candidate markers in the cells types was similar to those of the dataset GSE200997 (Supplementary Figure 4A, 4B).

**Table 5:** The differential expression of the marker genes in each cell type based on GSE14473.

**Markers**	**myAUC**	**avg_diff**	**power**	**avg_log2FC**	**pct.1**	**pct.2**	**celltypes**
HSPA1A	0.585	17.67964	0.17	0.909902001	0.38	0.24	T Cells
FOSB	0.262	6.571136	0.476	-0.97096937	0.46	0.78	B cells
RPL35	0.713	3.467668	0.426	0.668682238	0.976	0.919	B cells
CD69	0.692	34.30116	0.384	0.987759617	0.485	0.114	B cells
REG1A	0.449	-181.39	0.102	-1.06795157	0.004	0.105	Epithelial cells
TESC	0.344	-7.81038	0.312	-0.82064498	0.151	0.45	Epithelial cells
HSPA1A	0.699	5.855744	0.398	1.225526512	0.52	0.135	Epithelial cells
GDF15	0.319	-12.5721	0.362	-1.2395757	0.161	0.499	Epithelial cells
LGALS2	0.312	-21.5721	0.376	-1.59399592	0.141	0.487	Epithelial cells
C11orf96	0.397	-2.6086	0.206	-0.39939187	0.238	0.436	Stromal cells

Additionally, we investigated the expression differences of the candidate biomarkers in two bulk RNA-seq dataset. Based on the TCGA-COAD cohort, we observed that only REG1A and CD69 were significantly differential expressed between LCC and RCC patients (*P* <0.05, Figure 4H). In the dataset GSE103479, all the candidate markers were not significantly differential expressed between LCC and RCC patients (*P* > 0.05, Supplementary Figure 4C).

Based on the two bulk RNA-seq datasets, we exhibited the proportions of LCC and RCC patients in each CMS group (Figure 4I, 4J). We observed that *FOSB* and *GDF15* were significantly expressed among CMS groups in both the two bulk RNA-seq cohorts (*P* <0.05, Figure 4K and Supplementary Figure 4D).

### TME differences between LCC and RCC patients

**Figure 5:**
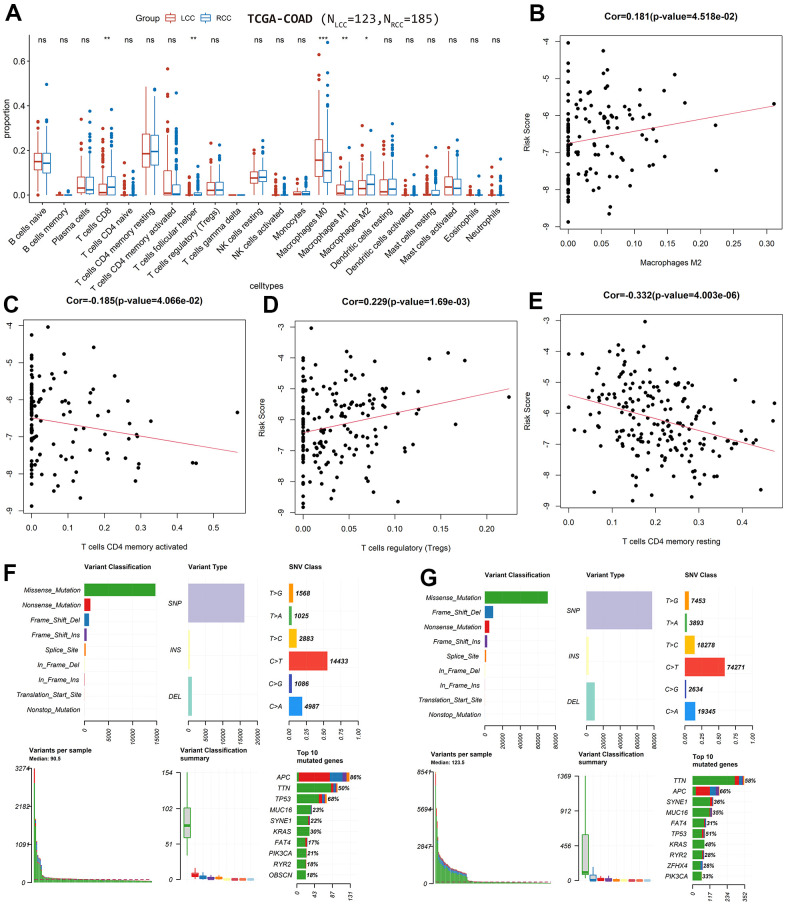
**Differences in immune-infiltrating and mutation profiles between LCC and RCC patients based on TCGA-COAD cohort.** (**A**) Differences in the composition of immune cell types between LCC and RCC patients. (**B**) The correlation between the immune-infiltrating degree of M2 macrophages with LCC patient risk scores. (**C**) The correlation between the immune-infiltrating degree of activated CD4 memory T cells with LCC patient risk scores. (**D**) The correlation between the immune-infiltrating degree of Tregs with RCC patient risk scores. (**E**) The correlation between the immune-infiltrating degree of resting CD4 memory T cells with RCC patient risk scores. (**F**) LCC patients’ mutation profiles. (**G**) RCC patients’ mutation profiles.

Based on the TCGA-COAD cohort, the proportions of 22 immune cell types were estimated in LCC and RCC patients using the CIBERSORT algorithm. The immune-infiltrating profiles of LCC and RCC patients are shown in Supplementary Figure 3A, 3B. When we compared the composition of immune cell types between LCC and RCC patients, we found significant differences in CD8 and regulatory (Tregs) T cells and M0, M1, and M2 macrophages (Figure 5A).

In the LCC prognostic model, the immune-infiltrating degree of M2 macrophages was positively correlated with risk score (*cor* = 0.181, *P* = 0.045; Figure 5B), while the immune-infiltrating degree of activated CD4 memory T cells was negatively correlated (*cor* = −0.185, *P* = 0.041; Figure 5C). In the RCC prognostic model, the immune-infiltrating degree of Tregs was positively correlated with risk score (*cor* = 0.229, *P* = 0.002; Figure 5D), while the immune-infiltrating degree of resting CD4 memory T cells was negatively correlated (*cor* = −0.332, *P* = 4.0×10^-6^; Figure 5E).

**Figure 6:**
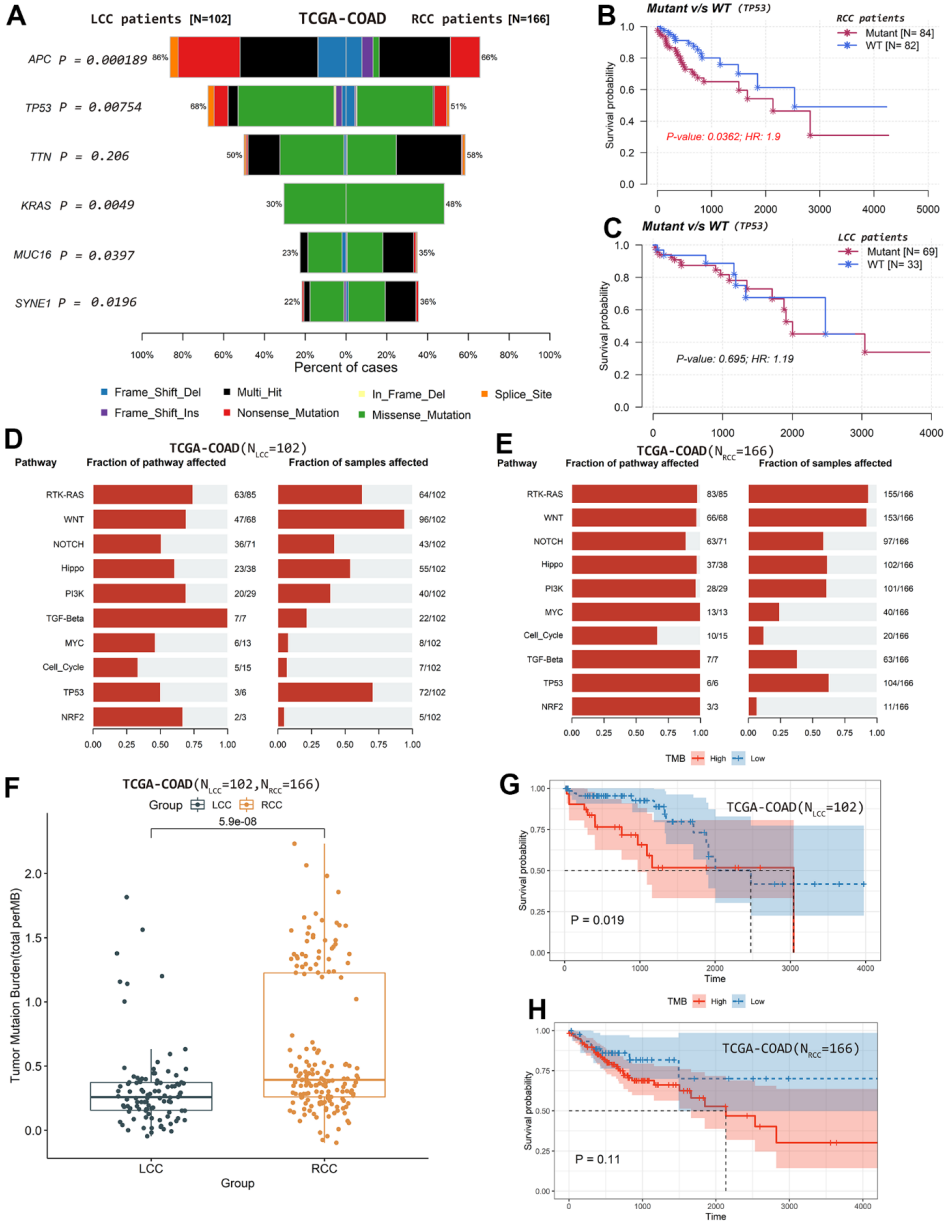
**Differences in mutation profiles and affected oncogenic pathways and their relationships with patient OS based on TCGA-COAD cohort.** (**A**) Differences in the top mutated genes. (**B**) *TP53* mutation status was significantly associated with OS in RCC patients. (**C**) *TP53* mutation status was not significantly associated with OS in LCC patients. (**D**) The fraction of pathways and samples affected by the oncogenic pathways in RCC patients. (**E**) The fraction of pathways and samples affected by the oncogenic pathways in LCC patients. (**F**) TMB values were significantly higher in RCC than in LCC patients. (**G**) LCC patients with lower TMB values had better OS. (**H**) TMB values were not significantly associated with OS in RCC patients.

Additionally, we plotted and compared the mutation profiles of LCC and RCC patients using the *maftools* R package. While the top 10 mutated genes in LCC and RCC patients were similar, their ranking differed. The median number of mutations in LCC patients was higher than in RCC patients. In LCC and RCC patients, the most common variant type was single nucleotide polymorphisms (SNP), the most common SNP classification was missense, and the most common SNP class was C>T (Figure 5F, 5G). Among the top 10 mutated genes, adenomatous polyposis coli regulator of WNT signaling pathway (*APC*) and tumor protein p53 (*TP53*) mutation rates were significantly higher in LCC patients (*P* < 0.05). In contrast, Kirsten rat sarcoma virus proto-oncogene GTPase (*KRAS*), spectrin repeat containing nuclear envelope protein 1 (*SYNE1*), and mucin 16 cell surface associated (*MUC16*) the mutation rates were significantly higher in RCC patients (*P* < 0.05; Figure 6A). Moreover, the mutation status of *TP53* was significantly associated with OS in RCC patients (*HR* = 1.9, *P* = 0.036; Figure 6B) but not in LCC patients (*HR* = 1.19, *P* = 0.695; Figure 6C). The mutation statuses of *APC*, *KRAS*, *SYNE1*, and *MUC16* were not significantly associated with OS in LCC or RCC patients (*P* >0.05; Supplementary Figure 5C–5J). Figure 6D, 6E shows that the fractions of pathways and samples affected by the oncogenic pathways in RCC patients were higher than in LCC patients.

We also calculated and visualized each sample’s TMB value. The median TMB value was 1.81/Mb in LCC patients (Supplementary Figure 5K) and 2.47/Mb in RCC patients (Supplementary Figure 5L). TMB values were significantly higher in RCC patients than in LCC patients (*P* = 5.9×10^-8^; Figure 6F). Moreover, LCC patients with lower TMB values had better OS (*P* = 0.019; Figure 6G). However, TMB values were not significantly associated with OS in RCC patients (*P* = 0.110; Figure 6H).

### Prediction and comparison of the drug response in LCC and RCC patients

**Figure 7:**
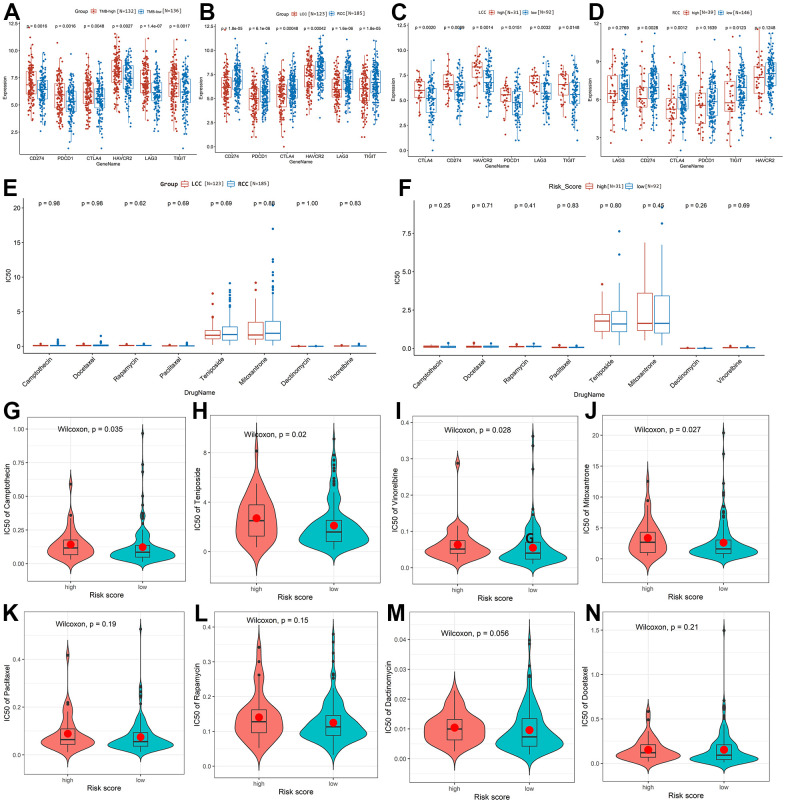
**Prediction and comparison of the drug response in LCC and RCC patients based on TCGA-COAD cohort.** (**A**) The expression of immune checkpoint targets was significantly higher in TMB-high patients than in TMB-low patients. (**B**) The expression of immune checkpoint targets was significantly higher in RCC patients than in LCC patients. (**C**) The expression of immune checkpoint targets was significantly higher in high-risk than low-risk LCC patients. (**D**) Comparison of immune checkpoint target expression between high-risk and low-risk RCC patients. (**E**) Sensitivity to the eight drugs did not differ significantly between LCC and RCC patients. (**F**) Drug response sensitivity did not differ significantly between high-risk and low-risk LCC patients. (**G**–**N**) Correlations between the average IC_50_ values of the eight drugs and RCC patient risk scores.

Figure 7A exhibited that the expression level of immune checkpoint targets in TMB-high group was significantly higher than that in TMB-low group (*P* < 0.05). And the expression of immune checkpoint targets in RCC patients was significantly higher than in LCC patients (*P* < 0.05; Figure 7B). Among LCC patients, the expression of immune checkpoint targets was significantly higher in the high-risk group than in the low-risk group, indicating that the high-risk group would be more sensitive to immunotherapy (*P* < 0.05, Figure 7C). Among RCC patients, cluster of differentiation 274 (*CD274*), cytotoxic T-lymphocyte associated protein 4 (*CTLA4*), and T cell immunoreceptor with Ig and ITIM domains (*TIGIT*) expression were significantly higher in the low-risk group than in the high-risk group. However, lymphocyte activating 3 (*LAG3*), programmed cell death 1 (*PDCD1*), and hepatitis A virus cellular receptor 2 (*HAVCR2*) expression did not differ significantly between the high- and low-risk groups (Figure 7D). Therefore, low-risk RCC patients are more likely to benefit from immunotherapy drugs targeting CD274, CTLA4, or TIGIT.

Additionally, we found that CC patients were sensitive to 44 drugs (average IC_50_ <5; Supplementary Table 4), of which eight were related to CC treatment. Figure 7E shows that sensitivity to these eight drugs does not differ significantly between LCC and RCC patients (*P* > 0.05). In addition, drug response sensitivity did not differ significantly between high-risk and low-risk LCC patients (*P* > 0.05, Figure 7F). However, the average IC_50_ values for camptothecin, teniposide, vinorelbine, and mitoxantrone were significantly lower in low-risk than in high-risk RCC patients, indicating that low-risk patients will be more sensitive to them (Figure 7G–7J). Sensitivity to the other four drugs did not differ significantly between high-risk and low-risk RCC patients (Figure 7K–7N).

## DISCUSSION

Extensive studies have identified various signatures for predicting CC prognosis, diagnosis, and treatment [21–23]. However, CC shows significant tumor-location-based differences, including phenotypic characteristics, TME, and treatment response [9, 24–26]. Consistent with a previous study [7], our study found that prognosis was significantly better in LCC than in RCC patients (Supplementary Figure 1A, 1B). To further understand the differences between LCC and RCC, we identified DEGs between LCC and RCC cells for each cell type based on scRNA-seq data.

The common prognostic models are mainly based on gene-sequencing dataset. For example, Liu et al. [27] found that nine ferroptosis-related long noncoding RNAs could be used as prognostic markers of COAD, and the prognostic signature has a good predictive value because of the AUC>0.8 for 5-year survival. However, our study built prognostic models for LCC or RCC patients through TCGA-COAD, where the 5-year OS AUCs for the LCC and RCC models were 0.926 and 0.836, respectively, suggesting that our prognostic models had better performance. In addition, similar to other study [28], the performance of the RCC model is worse than that of the LCC model. Besides, we also compared the expression differences of the identified markers in TCGA-COAD cohort (Figure 3H), and found that only *REG1A* and *CD16* were significantly differential expressed between LCC and RCC samples, which may indicate that DEGs obtained from bulk RNA-seq data cannot reflect the true expression of genes in different cell types. According the prognostic markers’ expression in each cell type (Table 4), the annotated eight main cell types could be categorized into 3 types:1) type 1(immune cells): T cells, B cells and macrophage; 2) type 2: epithelial cells; 3) type 3(stromal cells): fibroblasts, endothelial, and stem cells. It can be seen that the expression trend of the same gene in the same cell type is the same, but the expression trend is different in different cell types. For example, *HSPA1A* and *FOSB* are up-regulated in type 1 and down-regulated in type 3. *GDF15* is up-regulated in type 2 and down-regulated in type 3.

In the five-gene LCC prognostic model, low *C11orf96* and *FOSB* expression levels were associated with lower risk scores and longer survival time. In contrast, the high expression levels of *REG1A*, *RPL35*, and *TESC* were associated with higher risk scores and shorter survival time. These findings indicate that *C11orf96* and *FOSB* are risk factors, while *REG1A*, *RPL35*, and *TESC* are protective factors, which is consistent with the results shown in Figure 3B. A four-gene prognostic model was also developed for RCC patients, comprising *HSPA1A*, *CD69*, *GDF15*, and *LGALS2*. *HSPA1A* was a risk factor for RCC, while *CD69*, *GDF15*, and *LGALS2* were protective factors. *REG1A*, *RPL35*, *TESC*, *HSPA1A*, and *GDF15* have been associated with CC prognosis in all signatures [29–33].

Furthermore, we found many significant TME differences between LCC and RCC. First, while the infiltration degree of M0 macrophages was significantly higher in LCC patients than in RCC patients, the infiltration degrees of CD8 T cells and M1 macrophages were instead significantly lower. Secondly, while *APC* and *TP53* mutation rates were significantly lower in RCC patients than in LCC patients, *KRAS*, *SYNE1*, and *MUC16* mutation rates were instead significantly higher. In addition, TMB values were significantly higher in RCC patients than in LCC patients. These TME differences may cause differences in prognosis or treatment between LCC and RCC patients.

Additionally, consistent with the results found in previous study [5], patients in CMS1 subtype were mainly RCC, while CMS2 were mainly LCC (Figure 4I, 4J), which may indicate that CMS2 has better OS than CMS1 subtype. Otherwise, patients in TMB-high group had higher expression level of immune checkpoint targets than patients in TMB-low group, and the expression of immune checkpoint targets in RCC patients was significantly higher than in LCC patients, which may indicate that RCC patients were more likely to benefit from immunotherapy.

In summary, we systematically investigated differences in prognosis, TME, and treatment between RCC and LCC patients by integrative analysis of scRNA-seq and bulk RNA-seq data, providing insights into LCC and RCC heterogeneity. We also explored immunotherapy and drug chemotherapy differences between LCC and RCC patients, which may be conducive to providing patients with more accurate treatment.

## Supplementary Material

Supplementary Figures

Supplementary Table 1

Supplementary Table 2

Supplementary Tables 3 and 4
