# Supplementary material for: Comparative analyses of the prognosis, tumor immune microenvironment, and drug treatment response between left-sided and right-sided colon cancer by integrating scRNA-seq and bulk RNA-seq data
Source: Aging (Albany NY). 2023 Jul 24;15(14):7098–123. doi: 10.18632/aging.204894 (PMC10415577; doi:10.18632/aging.204894)
Supplement: Supplementary Figures [file aging-15-204894-s001.pdf]

SUPPLEMENTARY FIGURES

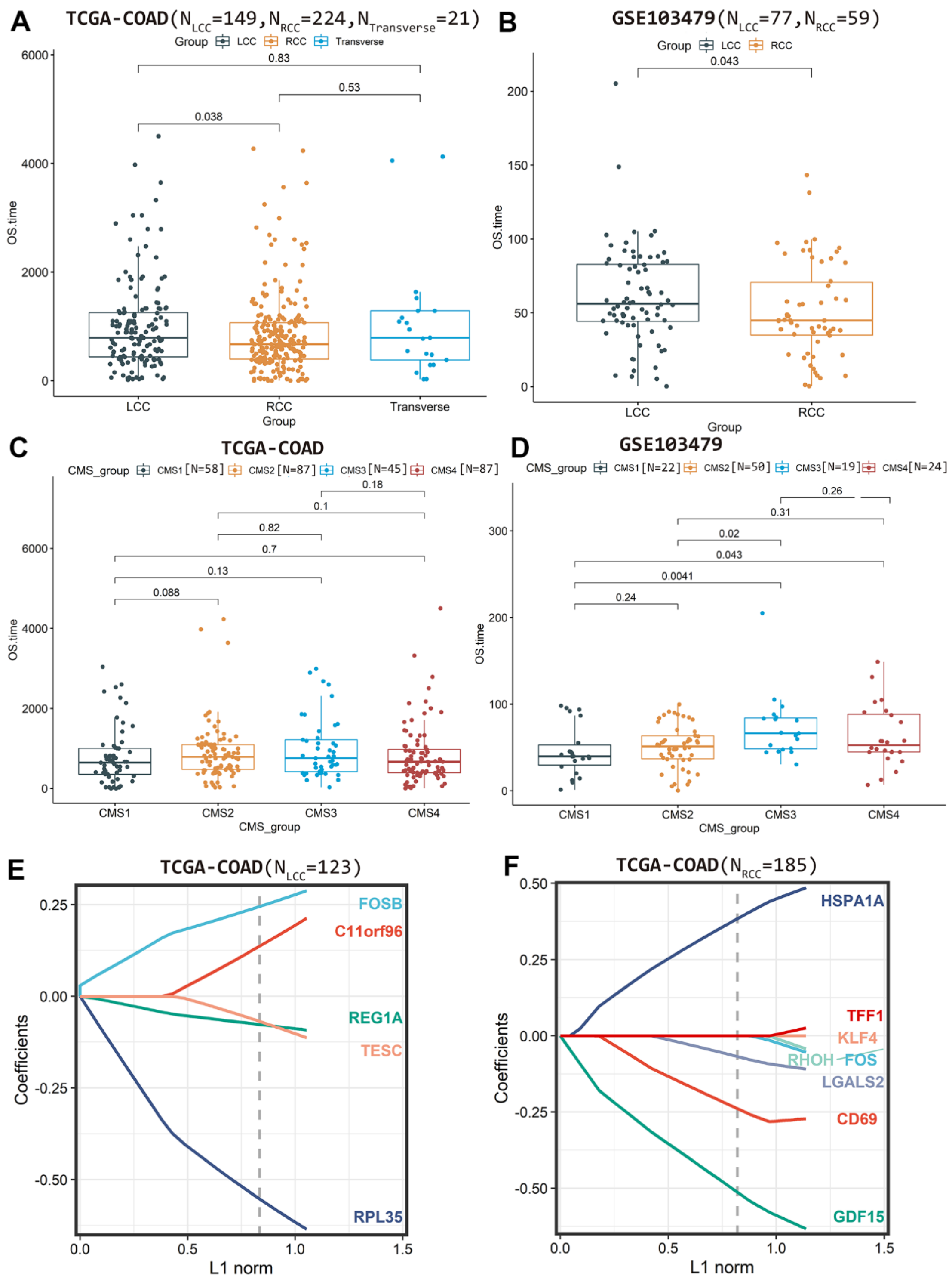

Supplementary Figure 1. Survival time comparison between patients with LCC and RCC, based on (**A**) TCGA-COAD cohort, (**B**) GSE103479 cohort. Survival time comparison between patients in CMS subtypes, based on (**C**) TCGA-COAD cohort, (**D**) GSE103479 cohort. Candidate prognostic signature coefficients for (**E**) LCC patients, (**F**) RCC patients based on TCGA-COAD cohort.

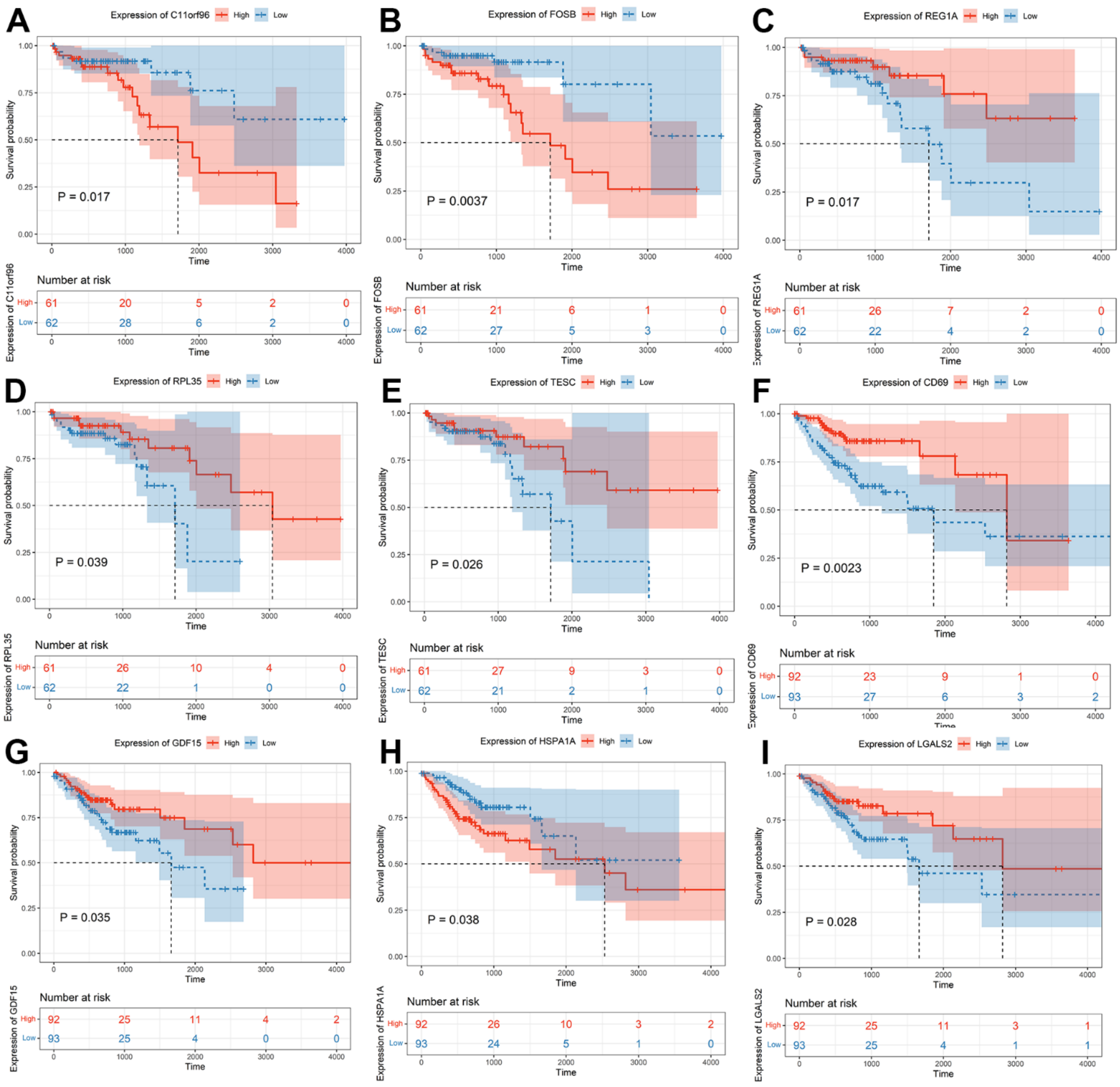

Supplementary Figure 2. The relationship between prognostic markers' expression with patients' OS, including (**A**) C11orf96, (**B**) FOSB, (**C**) REG1A, (**D**) RPL35, (**E**) TESC, (**F**) CD69, (**G**) GDF15, (**H**) HSPA1A, (**I**) LGALS2.

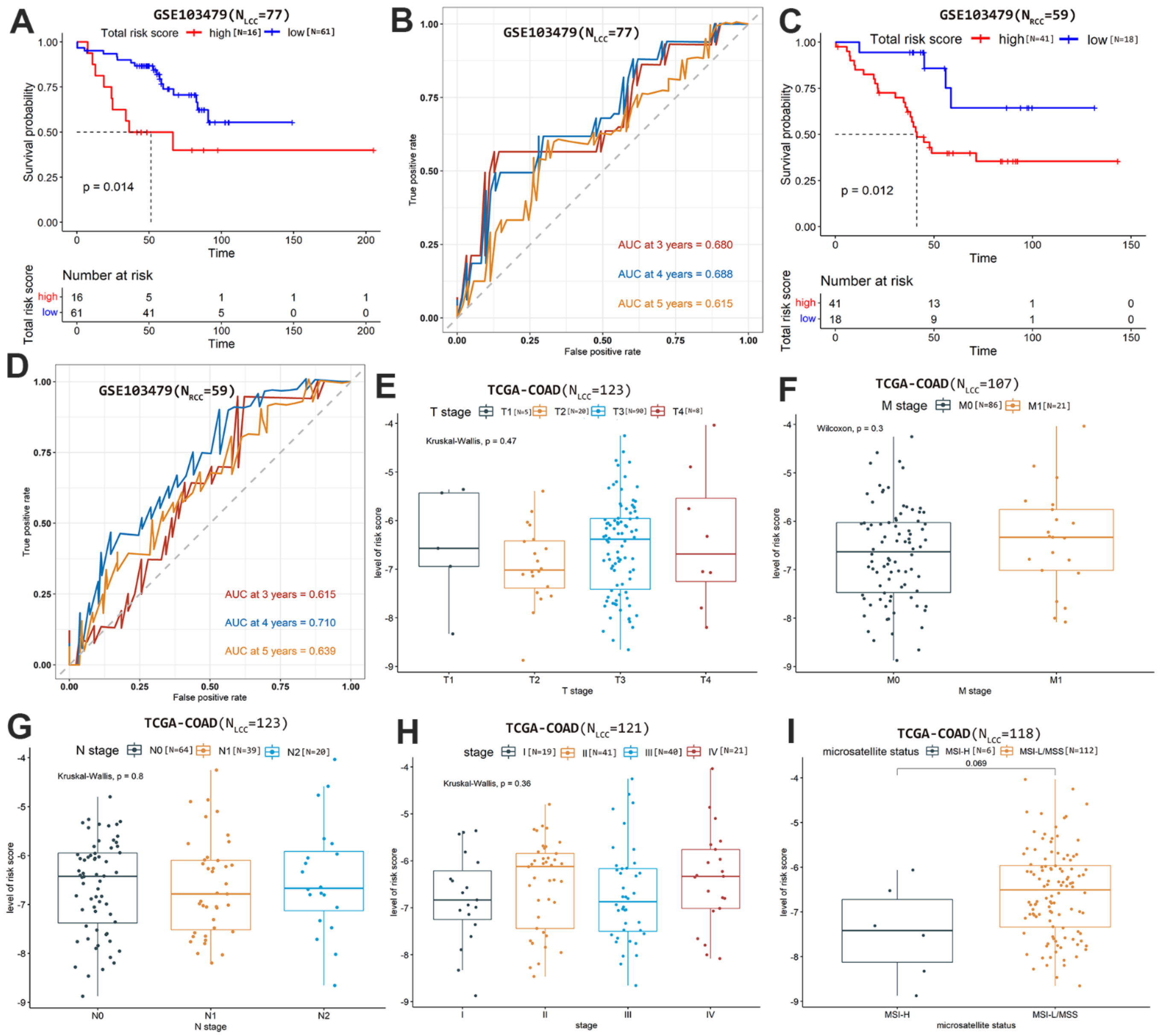

Supplementary Figure 3. Validation the identified prognostic markers based on GSE103479. (**A**) A Kaplan–Meier curve shows that low-risk score LCC patients had better OS than high-risk score LCC patients. (**B**) The AUCs of the prognostic model for LCC patients. (**C**) A Kaplan–Meier curve shows that low-risk score RCC patients had better OS than high-risk score RCC patients. (**D**) The AUCs of the prognostic model for RCC patients. The relationship between LCC patients' risk scores and clinical features, (**E**) T stage, (**F**) M stage, (**G**) N stage, (**H**) Advanced pathological stages, (**I**) Microsatellite status, based on TCGA-COAD cohort.

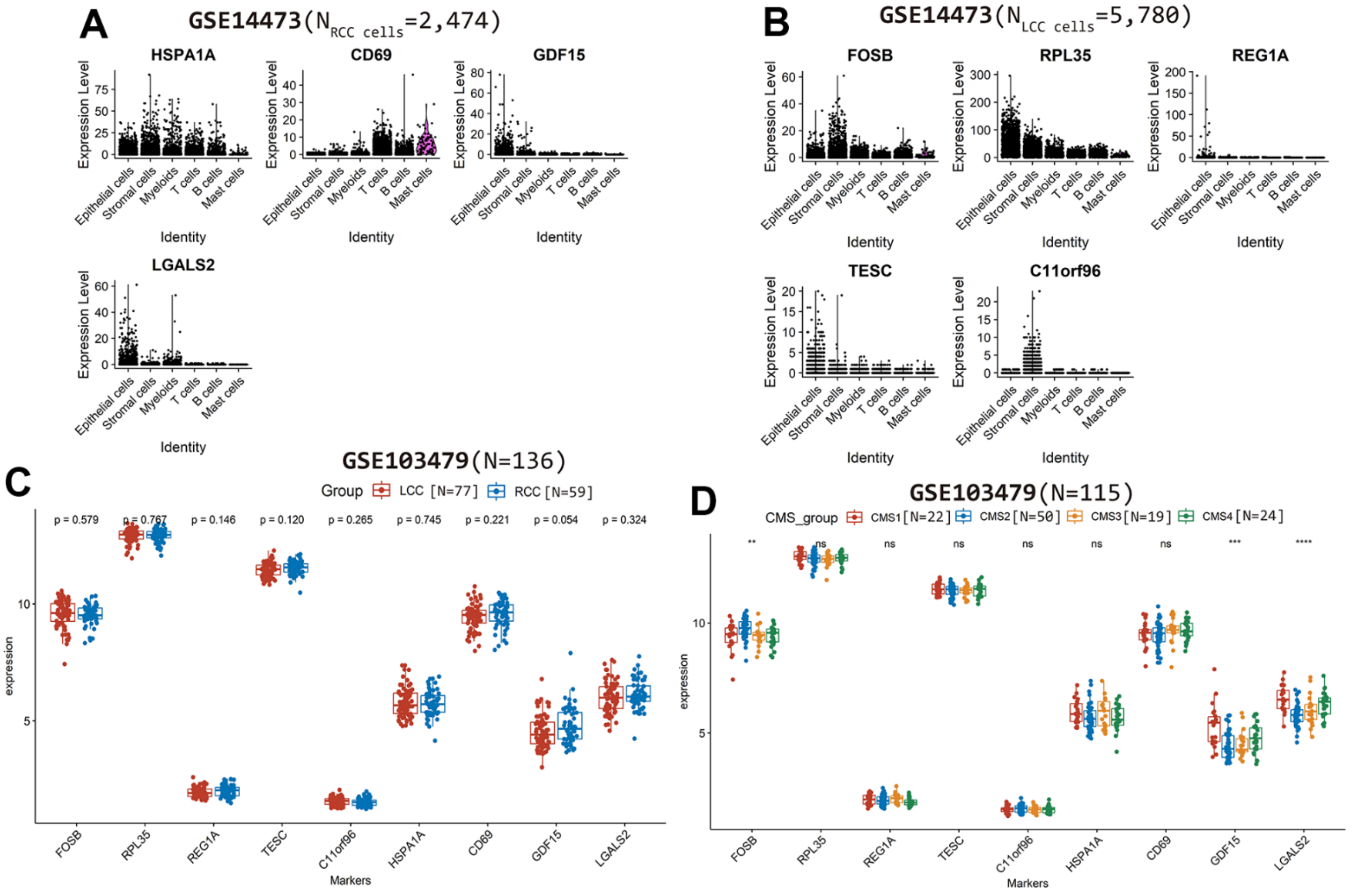

Supplementary Figure 4. The expression level of prognostic markers in each cell types based on scRNA-seq dataset GSE14473, such as (**A**) RCC cells and (**B**) LCC cells. (**C**) Comparing the expression differences of markers between LCC and RCC samples based on TCGA-COAD cohort. (**D**) Comparing the expression differences of markers among CMS subtypes based on TCGA-COAD cohort.

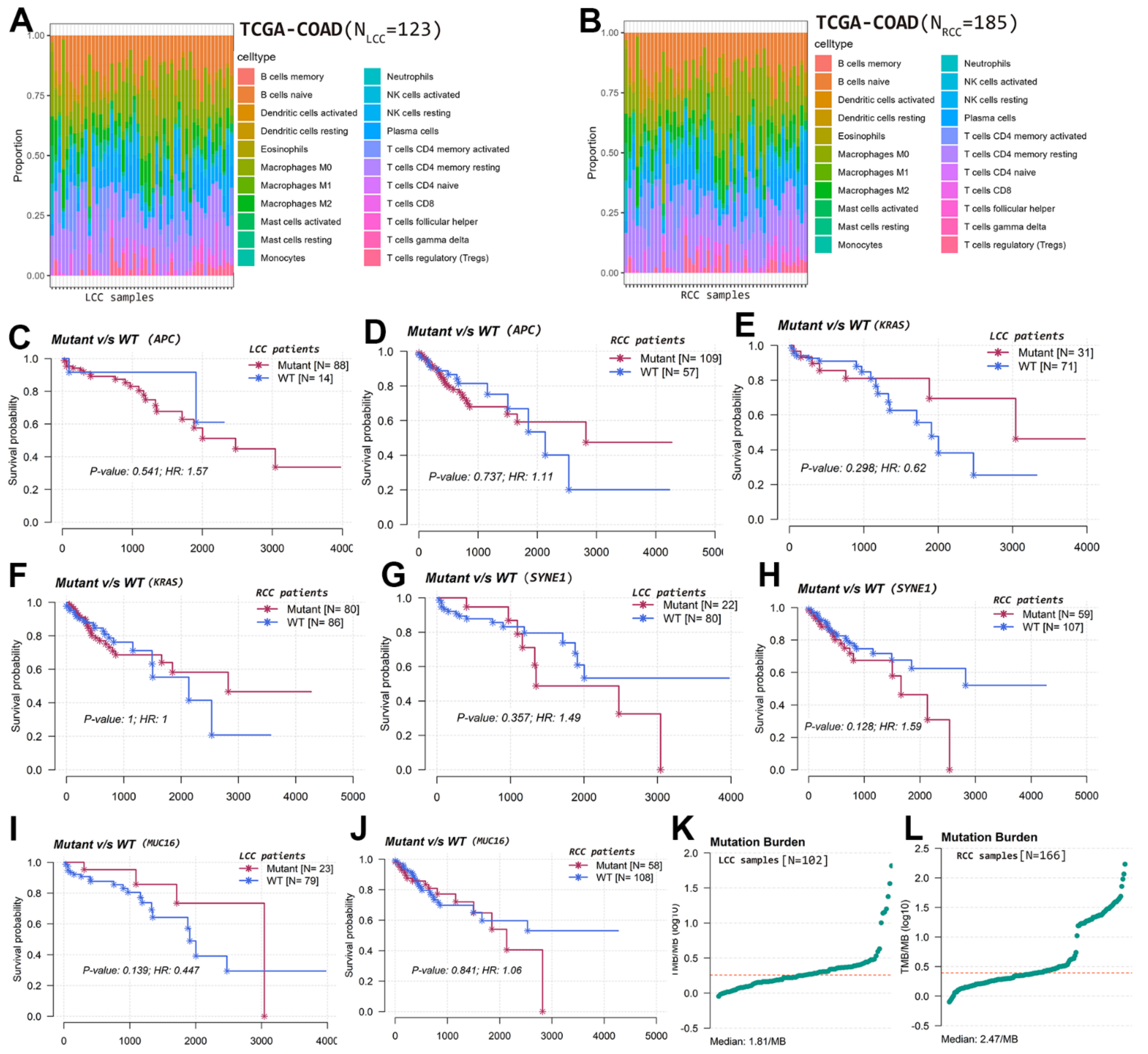

Supplementary Figure 5. The mutation profiles and relationships between the status of top mutated genes and OS. (**A**) LCC sample mutation profiles. (**B**) RCC sample mutation profiles. (**C**) Relationships between *APC* mutation status and OS in LCC patients. (**D**) Relationships between *APC* mutation status and OS in RCC patients. (**E**) Relationships between *KRAS* mutation status and OS in LCC patients. (**F**) Relationships between *KRAS* mutation status and OS in RCC patients. (**G**) Relationships between *SYNE1* mutation status and OS in LCC patients. (**H**) Relationships between *SYNE1* mutation status and OS in RCC patients. (**I**) Relationships between *MUC16* mutation status and OS in LCC patients. (**J**) Relationships between *MUC16* mutation status and OS in RCC patients. (**K**) The median TMB value in LCC patients. (**L**) The median TMB value in RCC patients.
