# Supplementary material for: Comparative analyses of the prognosis, tumor immune microenvironment, and drug treatment response between left-sided and right-sided colon cancer by integrating scRNA-seq and bulk RNA-seq data
Source: Aging (Albany NY). 2023 Jul 24;15(14):7098–123. doi: 10.18632/aging.204894 (PMC10415577; doi:10.18632/aging.204894)
Supplement: Supplementary Table 1 [file aging-15-204894-s002.pdf]

SUPPLEMENTARY TABLE

Supplementary Table 1. The detailed information of GSE103479.

	LCC (N=76)	RCC (N=58)	Overall (N=134)
**Gender**			
Female	30 (39.5%)	31 (53.4%)	61 (45.5%)
Male	46 (60.5%)	27 (46.6%)	73 (54.5%)
**Age**			
<=60	19 (25.0%)	4 (6.9%)	23 (17.2%)
>60	57 (75.0%)	54 (93.1%)	111 (82.8%)
**CMS**			
CMS1	6 (7.9%)	16 (27.6%)	22 (16.4%)
CMS2	33 (43.4%)	17 (29.3%)	50 (37.3%)
CMS3	13 (17.1%)	6 (10.3%)	19 (14.2%)
CMS4	14 (18.4%)	10 (17.2%)	24 (17.9%)
Unknown	10 (13.2%)	9 (15.5%)	19 (14.2%)
**stage**			
II	41 (53.9%)	28 (48.3%)	69 (51.5%)
III	35 (46.1%)	30 (51.7%)	65 (48.5%)
**OS**			
Alive	51 (67.1%)	31 (53.4%)	82 (61.2%)
Death	25 (32.9%)	27 (46.6%)	52 (38.8%)
**OS.time**			
Mean (SD)	60.7 (32.8)	51.1 (32.1)	56.6 (32.7)
Median [Min, Max]	56.1 [0.329, 205]	44.8 [0.362, 143]	51.2 [0.329, 205]
**T**			
T1	1 (1.3%)	0 (0%)	1 (0.7%)
T2	4 (5.3%)	2 (3.4%)	6 (4.5%)
T3	54 (71.1%)	38 (65.5%)	92 (68.7%)
T4	17 (22.4%)	18 (31.0%)	35 (26.1%)
**M**			
M	38 (50.0%)	27 (46.6%)	65 (48.5%)
M0	38 (50.0%)	31 (53.4%)	69 (51.5%)
**N**			
N0	41 (53.9%)	28 (48.3%)	69 (51.5%)
N1	23 (30.3%)	24 (41.4%)	47 (35.1%)
N2	12 (15.8%)	6 (10.3%)	18 (13.4%)
