# Supplementary material for: Comparative analyses of the prognosis, tumor immune microenvironment, and drug treatment response between left-sided and right-sided colon cancer by integrating scRNA-seq and bulk RNA-seq data
Source: Aging (Albany NY). 2023 Jul 24;15(14):7098–123. doi: 10.18632/aging.204894 (PMC10415577; doi:10.18632/aging.204894)
Supplement: Supplementary Tables 3 and 4 [file aging-15-204894-s004.pdf]

Supplementary Table 3. The detailed information of the 37 prognostic genes.

Type	GeneName	P-value
RCC	HSPA1A	0.038
	ZFP36	0.011
	FOS	0.031
	BTG1	0.015
	CD69	0.0023
	SARAF	0.042
	RHOH	0.0068
	IGKV3-20	0.024
	IGLC2	0.012
	IGLV3-1	0.018
	IGLC3	0.036
	KLF4	0.009
	TFF1	0.01
	GDF15	0.035
	LGALS2	0.028
	TNFSF11	0.013
	CXCL2	0.033
	PRKAR2B	0.038
	FSTL3	0.03
LCC	GSTM1	0.038
	GADD45B	0.039
	DUSP1	0.024
	FOSB	0.0037
	SLC2A3	0.0091
	CLEC2B	0.025
	RPL35	0.039
	REG1A	0.017
	TESC	0.026
	MUC2	0.023
	UCP2	0.036
	BCL3	0.046
	C11orf96	0.017

Supplementary Table 4. The detailed information of the 44 sensitive drugs.

DrugName	Average IC50 of LCC	Average IC50 of RCC
AZD7762_1022	1.1204019813473	1.14668122516778
AZD8055_1059	0.819746194430271	0.817151413765119
BI-2536_1086	1.43477406540067	1.44516285070571
BMS-754807_2171	2.18563326304696	2.0995544101959
Bortezomib_1191	0.0079553655585555	0.00795034351693453
Buparlisib_1873	2.64067623292989	2.71915407159633
Camptothecin_1003	0.106941939328332	0.126685792093699
CDK9_5038_1709	0.101784844963055	0.110910831372088
CDK9_5576_1708	0.697126165684043	0.747710358035258
Dactinomycin_1811	0.0914565247765287	0.0947628168703208
Dactinomycin_1911	0.00930230788904291	0.00979743548734258
Dactolisib_1057	0.208373502951644	0.217767507782637
Daporinad_1248	0.0159589729625909	0.0152438928452886
Dihydrorotenone_1827	2.67874699895989	2.58263284297451
Dinaciclib_1180	0.0622924469820657	0.0643635837037069
Docetaxel_1007	0.0114575094281596	0.0123631384452993
Docetaxel_1819	0.115262401841707	0.153395306777572
Eg5_9814_1712	0.0514518409829225	0.0578239198162486
Epirubicin_1511	0.39873780453285	0.443820929049132
Foretinib_2040	2.8036548807378	2.8351526305029
Gemcitabine_1190	0.670396146804438	0.885694591519111
GNE-317_1926	1.7357221893917	1.78365491687438
Luminespib_1559	0.114542509427635	0.124908217906364
MG-132_1862	0.199904631860733	0.200702148062894
Mitoxantrone_1810	2.35472957002234	2.78548918493597
MK-1775_1179	1.84196464279701	1.88729184406175
Obatoclax Mesylate_1068	4.21859659918443	4.25533904583997
Paclitaxel_1080	0.0699157080248199	0.0776069245152012
PD0325901_1060	1.78951162837727	1.81467908936507
Pevonedistat_1529	2.2223919772503	2.58925495765451
Pictilisib_1058	4.23695684306298	4.34822145911636
Podophyllotoxin bromide_1825	0.534256310699056	0.584762508793234
Rapamycin_1084	0.124666540178059	0.128443408563328
Sabutoclax_1849	0.715483989210096	0.735267805242988
Sepantronium bromide_1941	0.0141875929157305	0.0148052970774564
Staurosporine_1034	0.0530226514569955	0.0570266509673851
Telomerase Inhibitor IX_1930	1.74804204176677	1.80528995955417
Teniposide_1809	1.88461723681019	2.23104661366957
Topotecan_1808	1.23814143201278	1.36328885227713
Trametinib_1372	2.01427331557814	2.10670533866479
Vinblastine_1004	0.0258534037328264	0.0307996754635948
Vincristine_1818	0.19105207223191	0.240791887001131
Vinorelbine_2048	0.0490856376646081	0.0567394338636524
Vorinostat_1012	4.29191225842706	4.32462213402817
